# FEPI-MB: identifying SNPs-disease association using a Markov Blanket-based approach

**DOI:** 10.1186/1471-2105-12-S12-S3

**Published:** 2011-11-24

**Authors:** Bing Han, Xue-wen Chen, Zohreh Talebizadeh

**Affiliations:** 1 Bioinformatics and Computational Life Sciences Laboratory, Information and Telecommunication Technology Center, Department of Electrical Engineering and Computer Science, The University of Kansas, 1520 West 15th Street, Lawrence, KS 66045, USA; 2 Children’s Mercy Hospital and University of Missouri - Kansas City, Kansas City, MO 64108, USA

## Abstract

**Background:**

The interactions among genetic factors related to diseases are called epistasis. With the availability of genotyped data from genome-wide association studies, it is now possible to computationally unravel epistasis related to the susceptibility to common complex human diseases such as asthma, diabetes, and hypertension. However, the difficulties of detecting epistatic interaction arose from the large number of genetic factors and the enormous size of possible combinations of genetic factors. Most computational methods to detect epistatic interactions are predictor-based methods and can not find true causal factor elements. Moreover, they are both time-consuming and sample-consuming.

**Results:**

We propose a new and fast Markov Blanket-based method, FEPI-MB (Fast EPistatic Interactions detection using Markov Blanket), for epistatic interactions detection. The Markov Blanket is a minimal set of variables that can completely shield the target variable from all other variables. Learning of Markov blankets can be used to detect epistatic interactions by a heuristic search for a minimal set of SNPs, which may cause the disease. Experimental results on both simulated data sets and a real data set demonstrate that FEPI-MB significantly outperforms other existing methods and is capable of finding SNPs that have a strong association with common diseases.

**Conclusions:**

FEPI-MB algorithm outperforms other computational methods for detection of epistatic interactions in terms of both the power and sample-efficiency. Moreover, compared to other Markov Blanket learning methods, FEPI-MB is more time-efficient and achieves a better performance.

## Background

In recent years, the success of GWAS (genome-wide association studies) makes it possible to detect genetic factors that influence the susceptibility to particular diseases in human populations [1]. While most of GWAS search for one single contributing locus at a time, they fail to identify the combinational effect (epistasis) of genetic variants (i.e., single-nucleotide polymorphisms, or SNPs) associated with common complex diseases such as asthma, diabetes, and hypertension [2]. It is well known that epistatic interactions, not individual variant, are critical in unravelling genetic causes of complex human diseases [3]. However, the number of possible combinations of SNPs in a genome is enormous, which is infeasible to be evaluated exhaustively by experimental methods. Therefore, researchers resort to computational methods to detect epistatic interactions based on the genotyped data [2,4].

Recently, a number of statistical methods have been proposed to detect epistatic interactions. Among these methods, the most commonly used one is logistic regression (LR) [5]. However, logistic regression may not be appropriate for epistasis due to its overfitting problem due to the fact that the number of parameters will be much larger than the available samples. To avoid this shortcoming, Ritchie *et al*. proposed MDR (multifactor dimensionality reduction) [6,7], which utilizes the ratio of the number of cases to the number of controls in cells of risk table to reduce the dimensionality to one and select SNP combinations that have the highest prediction performance. The process of labelling each cell of risk table as “high risk” or “low risk” is a process of estimating parameters, which may also result in the overfitting problems when the size of SNP combinations is large. Furthermore, MDR selects the SNP combinations purely by the prediction performance and thus, it may not find true causal factors. Park and Hastie proposed the stepwise-penalized logistic regression (stepPLR) to overcome the drawbacks of logistic regression and MDR [8]. StepPLR makes some simple modifications for standard logistic regression (LR). For example, stepPLR combines the LR criterion with a penalization of the L2-norm of the coefficients. This modification makes stepPLR more robust to high-order epistatic interactions. Despite its modifications, stepPLR is time-consuming when estimating parameters, which is one essential limitation of regression methods. BEAM is a Bayesian marker partition model using Markov Chain Monte Carlo to reach an optimal marker partition and a new B statistic to check each marker or set of markers for significant associations [9]. Note that most statistical methods can not be applied to genome-wide analysis directly due to their computational complexity. The alternative approaches to parametric statistical methods are machine learning methods including Support Vector Machine (SVM) [10] and Random Forest [11]. Machine learning methods consider detecting epistatic interactions as a feature selection problem [12] and try to find the best combination of SNPs with the highest prediction accuracy of disease status. Therefore, Chen *et al*. test three feature selection method: RFE (recursive feature elimination), RFA (recursive feature addition), and GA (genetic algorithm) in [10] and Jiang *et al*. perform a greedy search in [11]. Like MDR, machine learning methods select SNPs based on classification/prediction accuracy and can not find true causal factors for disease. Moreover, machine learning-based methods tend to introduce many false positives because using more SNPs tends to produce higher classification accuracies.

**Figure 1:**
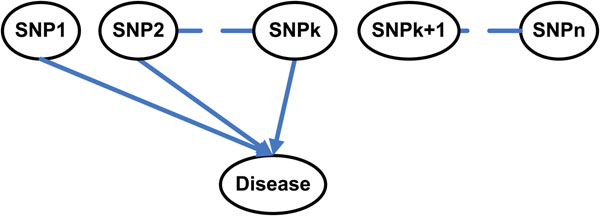
**Example of genome-wide association studies (GWAS).** The goal of genome-wide association studies is to identify the *k*-way interaction among disease SNPs: SNP1, SNP2, …, SNPk.

In this paper, we propose a new and fast Markov Blanket method, FEPI-MB (Fast EPistatic Interactions detection using Markov Blanket), to detect epistatic interactions. The Markov Blanket is a minimal set of variables, which can completely shield the target variable from all other variables. As shown in Figure 1, genome-wide association studies try to identify the *k*-way interaction among disease SNPs: SNP1, SNP2,…,SNPk and exclude all other unrelated normal SNPs (SNPk+1,…,SNPn). Thus, the Markov Blanket learning method is suitable for detection of epistatic interactions in genome-wide case-control studies, e.g., to identify a minimal set of SNPs which may cause the disease and require further experiments. Meanwhile the detected minimal set of causal SNPs can shield the disease from all other normal SNPs to decrease the false positive rate and reduce the cost of future validation experiments. Furthermore, Markov Blanket method performs a heuristic search by calculating the association between variables to avoid the time-consuming training process as in SVM and Random Forest.

Some Markov Blanket methods take a divide-and-conquer approach that breaks the problem of identifying Markov Blanket of variable T (MB (T)) into two subproblems: first, identifying parents and children of T (PC (T)) and, second, identifying the parents of the children of T (spouse). The goal of epistatic interactions detection is to identify causal interacting genes or SNPs for some certain diseases and therefore it is a special application of Markov Blanket method because we only need to detect the parents of the target variable T (disease status labels). Our new Markov Blanket method makes some simplifications to adapt to this special condition.

We apply the FEPI-MB algorithm to simulated datasets based on four disease models and a real dataset (the Age-related Macular Degeneration (AMD) dataset). We demonstrate that the proposed method significantly outperforms other commonly-used methods and is capable of finding SNPs strongly associated with diseases. Comparing to other Markov Blanket learning methods, our method is faster and can still achieve a better performance.

## Results

### Simulated data generation

We first evaluate the proposed FEPI-MB on simulated data sets, which are generated from three commonly used two-locus epistatic models [5,9] and one three-locus epistatic model developed [9]. Table 1 lists the disease odds for these four epistatic models, where *α* is the baseline effect and *θ* is the genotypic effect. Assume that an individual has genotype *g_A_* at locus A and genotype *g_B_* at locus B in a two-locus epistatic model, then the disease odds are(1)

**Table 1:** Four disease models

Model1	AA	Aa	aa
BB	*α*	*α*(1 + *θ*)	*α*(1 + *θ*)^2^
Bb	*α*(1 + *θ*)	*α*(1 + *θ*)^2^	*α*(1 + *θ*)^3^
bb	*α*(1 + *θ*)^2^	*α*(1 + *θ*)^3^	*α*(1 + *θ*)^4^

Model2	AA	Aa	aa

BB	*α*	*α*	*α*
Bb	*α*	*α*(1 + *θ*)	*α*(1 + *θ*)^2^
bb	*α*	*α*(1 + *θ*)^2^	*α*(1 + *θ*)^4^

Model3	AA	Aa	aa

BB	*α*	*α*	*α*
Bb	*α*	*α*(1 + *θ*)	*α*(1 + *θ*)
bb	*α*	*α*(1 + *θ*)	*α*(1 + *θ*)

Model4	AA		
	
	BB	Bb	bb

CC	*α*	*α*	*α*

Cc	*α*	*α*	*α*(1 + *θ*)
cc	*α*	*α*(1 + *θ*)	*α*
	Aa		

	BB	Bb	bb

CC	*α*	*α*	*α*(1 + *θ*)
Cc	*α*	*α*(1 + *θ*)	*α*
cc	*α*(1 + *θ*)	*α*	*α*

	aa		
	
	BB	Bb	bb

CC	*α*	*α*(1 + *θ*)	*α*
Cc	*α*(1 + *θ*)	*α*	*α*
cc	*α*	*α*	*α*

where *p*(*D*|*g_A_*,*g_B_*) is the probability that an individual has the disease given genotype (*g_A_*,*g_B_*) and  is the probability that an individual does not have the disease given genotype (*g_A_*,*g_B_*).

In Model1 the odds of disease increase in a multiplicative mode both within and between two loci. For example, an individual with Aa at locus A has larger odds, which are 1 + *θ* times relative to those of an individual who is homozygous AA; the aa homozygote has further increased disease odds by (1 + *θ*)^2^. We can also find similar effects on locus B. Finally the odds of disease for each combination of genotypes at loci A and B can be obtained by the product of the two within-locus effects. Model2 demonstrates two-locus interaction multiplicative effects because at least one disease-associated allele must be present at each locus to increase the odds beyond the baseline level. Moreover the increment of the disease-associated allele at loci A or B can further increase the disease odds by the multiplicative factor 1 + *θ*. Model3 specifies two-locus interaction threshold effects. Like Model 2, Model3 also requires at least one copy of the disease-associated alleles at both loci A and B. However the increment of the disease-associated allele does not increase the risk further. We call this as disease threshold effect. It means that a single copy of the disease-associated allele at each locus is required to increase odds of disease and this is the disease threshold. But after the disease threshold has already been met, having both copies of the disease-associated allele at either locus has no additional influence on disease odds. There are three disease loci in model 4. Some certain genotype combinations can increase disease risk and there are almost no marginal effects for each disease locus. Model 4 is more complex than Models 1, 2 and 3. All these four models are non-additive models and they differ in the way that the number of disease-associated allele increases the odds of disease. The prevalence of a disease is the proportion the total number of cases of the disease in the population and we assume that the disease prevalence is 0.1 for all these four disease models [9].

To generate data, we need to determine three parameters associated with each model: the marginal effect of each disease locus (*λ*), the minor allele frequencies (MAF) of both disease loci, and the strength of linkage disequilibrium (LD) between the unobserved disease locus and a genotyped locus [5]. LD is a nonrandom association of alleles at different loci and is quantified by the squared correlation coefficient *r*^2^ calculated from allele frequencies [5]. In this paper, we set *λ* equal to 0.3, 0.3, and 0.6 for models 1, 2, and 3, respectively. For model 4, we set *θ* = 7 arbitrarily because there are almost no marginal effects in model 4. We let MAF take four values (0.05, 0.1, 0.2, and 0.5) and let *r*^2^ take two values (0.7, 1.0) for each model. For each non-disease marker we randomly chose its MAF from a uniform distribution in [0.0. 0.5]. We first generate 50 small datasets and each dataset contains 100 markers genotyped for 1,000 cases and 1,000 controls based on each parameter setting for each model. To test the scalability of FEPI-MB, we also generate 50 large datasets and each dataset contains 500 markers genotyped for 2,000 cases and 2,000 controls using the same parameter setting for each model.

### Epistasis detection on simulated data

We compare the FEPI-MB algorithm with three commonly-used methods: BEAM, SVM and MDR on the four simulated disease models. To measure the performance of each method, we use “power” as the criterion function. Power is calculated as the fraction of 50 simulated datasets in which disease associated markers are identified and demonstrate statistically significant associations (*G*^2^ test values below a threshold for FEPI-MB) with the disease [9,11]. The BEAM software is downloaded from http://www.fas.harv-ard.edu/~junliu/BEAM and we set the threshold of the B statistic as 0.1 [9]. For SVM, we use LIBSVM with a RBF kernel to detect epistatic interactions and the same searching strategy as shown in [13]. Since MDR algorithm can not be applied to a large dataset directly, we first reduce the number of SNPs to 10 by ReliefF [14], a commonly-used feature selection algorithm, and then MDR performs an exhaustive search for a SNP set that can maximize cross-validation consistency and prediction accuracy. For the large datasets containing 500 markers genotyped for 2,000 cases and 2,000 controls, we only compare the performance of FEPI-MB, BEAM and SVM because ReliefF [14] in MDR can not work for large datasets of this scale.

**Figure 2:**
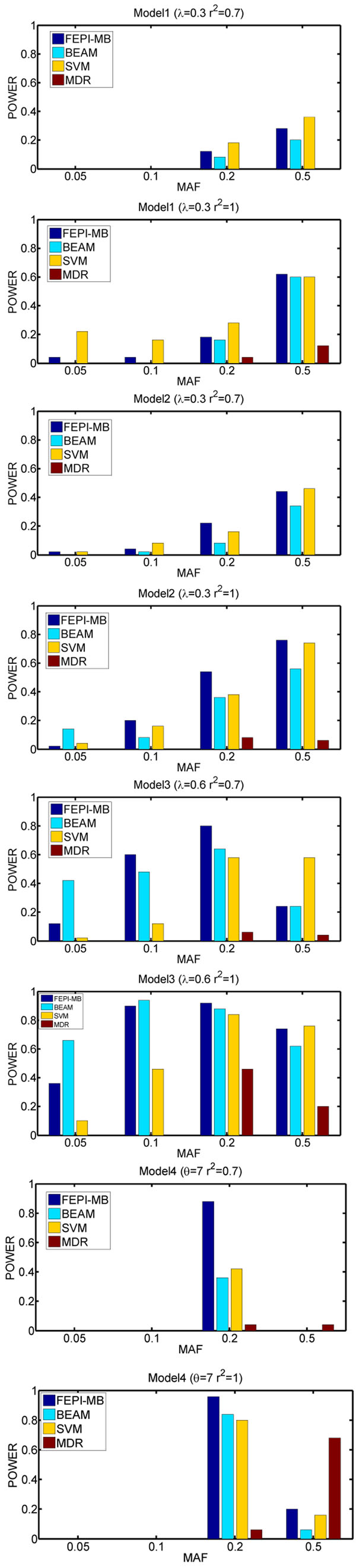
Performance comparison for small datasets containing 100 markers genotyped from 1000 cases and 1000 controls.

**Figure 3:**
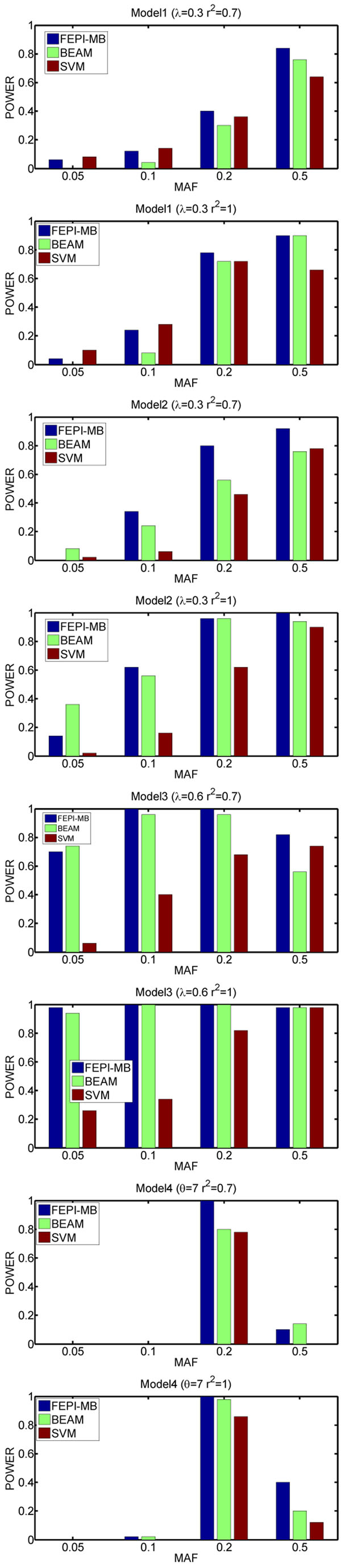
Performance comparison for large datasets containing 500 markers genotyped from 2000 cases and 2000 controls.

We show the results on the simulated data in Figures 2 and 3. As can be seen, FEPI-MB performs the best comparing to other three methods. BEAM is the second best. In most cases, the powers of MDR are much smaller than these of the FEPI-MB and BEAM algorithms. For the MDR algorithm, the poor performance may be due to the use of ReliefF to reduce SNPs from a very large dimensionality. We try another comparison experiment based on the simulated data containing only 40 markers, which makes us be able to apply MDR to the simulated data directly. The performance of MDR is still poor and this indicates that perhaps using the risk table as a predictor to detect epistatic interactions is not a good choice. In some cases, SVM can achieve a comparable or even better performance than FEPI-MB and BEAM, however, at the cost of introducing more false positives. Figure 3 also demonstrates the scalability of FEPI-MB on large datasets.

**Figure 4:**
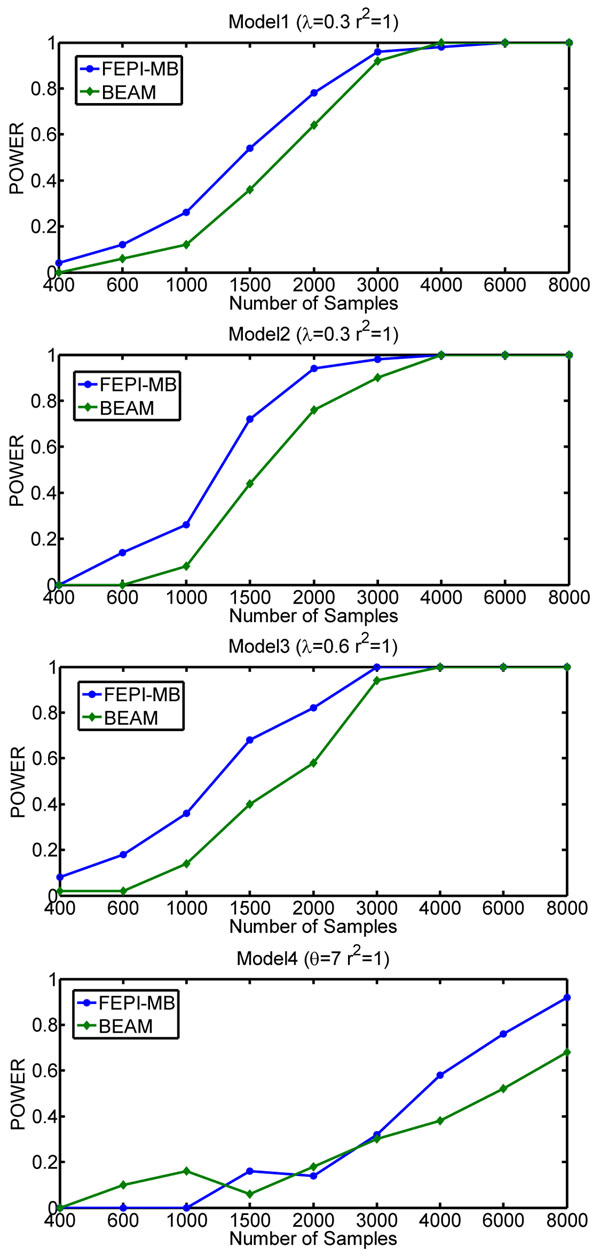
Effect of number of samples on the performance of FEPI-MB and BEAM.

An important issue for epistatic interaction detection in genome-wide association studies is the number of available samples. Typically, the size of samples is limited and consequently, computational model behaves differently. We explore the effect of the number of samples on the performance of BEAM and FEPI-MB (SVM will always introduce a large number of false positives and thus, is not compared here). We generate synthetic datasets containing 40 markers genotyped for different number of cases and controls with *r*^2^ = 1 and MAF=0.5. The result is shown in Figure 4 and we find that FEPI-MB can achieve a higher power than BEAM when the number of samples is the same in most cases. On the other hand, FEPI-MB needs fewer samples to reach the perfect power comparing to BEAM. So we can conclude that FEPI-MB is more sample-efficient than BEAM.

We also compare the performance of FEPI-MB with interIAMBnPC based on the large dataset from model1 to show the time efficiency of FEPI-MB. Among the three variants of IAMB, interIAMBnPC can achieve the best performance [15]. Both FEPI-MB and interIAMBnPC are written in MATLAB and all the experiments are run on an Intel Core 2 Duo T6600 2.20 GHz, 4GB RAM and Windows Vista. The results are shown in Table 2. As seen, FEPI-MB runs more than ten times faster than interIAMBnPC.

**Table 2:** Comparison of performance of FEPI-MB and interIAMBnPC for the large datasets of Model1

Model	*λ*	*r*^2^	MAF	Algorithm	Power	Average time (s)
1	0.3	0.7	0.05	FEPI-MB	3	0.4574
				interIAMBnPC	3	7.5505
			
			0.1	FEPI-MB	6	0.4437
				interIAMBnPC	5	9.2449
			
			0.2	FEPI-MB	20	0.4436
				interIAMBnPC	20	9.4295
			
			0.5	FEPI-MB	42	0.4449
				interIAMBnPC	42	8.2823
		
		1	0.05	FEPI-MB	2	0.4393
				interIAMBnPC	2	7.3610
			
			0.1	FEPI-MB	12	0.4421
				interIAMBnPC	12	9.7156
			
			0.2	FEPI-MB	39	0.4431
				interIAMBnPC	38	9.6498
			
			0.5	FEPI-MB	45	0.4449
				interIAMBnPC	43	9.1229

### Epistasis detection on AMD data

FEPI-MB demonstrates its greater power, sample-efficiency, and time-efficiency on simulated data with the number of SNPs less than 500. In practical problems, a typical GWAS genotype dataset contains at least more than 30,000 common SNPs. FEPI-MB can also be scalable to large-scale datasets in real genome-wide case-control studies. We apply FEPI-MB to an Age-related Macular Degeneration (AMD) dataset, which contains 116,204 SNPs genotyped with 96 cases and 50 controls [16]. AMD (OMIM 603075) [17] is a common genetic disease related to the progressive visual dysfunction in age over 70 in the developed country. We use the same preprocessing method as in [9,16]. After filtering, there are 97,327 SNPs lying in 22 autosomal chromosomes remained.

The searching time of FEPI-MB for AMD-related SNPs on an Intel Core 2 Duo T6600 2.20 GHz, 4GB RAM and Windows Vista is 96.4s and FEPI-MB detects two associated SNPs: rs380390 and rs2402053, which have a *G*^2^ test p-value of 5.36*10^-10^. The first SNP, rs380390, is already found in [16] with a significant association with AMD. The other SNP detected by the FEPI-MB algorithm is SNP rs2402053, which is intergenic between TFEC and TES in chromosome 7q31 [18].

It is worth noting that several lines of evidence have previously shown the long arm of 7q harbors genes implicated in retinal disorders. Among which is mapping of a locus on 7q31-q32 for retinitis pigmentosa, another retinal disease [19]. Ocular abnormalities have been reported for an individual with terminal deletion of chromosome 7q [20]. Mutations in a gene located at 7q32 have been reported in families with autosomal dominant retinitis pigmentosa [21]. More recently, Next-generation sequencing revealed mutations in another gene located on chromosome 7q31 in patients with a form of retinopathy [22].

The rs2402053 SNP identified in our study does not locate in any of the previously reported implicated genes in retinal disorders. Therefore, it may shed light on discovering a new genetic factor, on chromosome 7q, contributing to the underlying mechanism of AMD, a complex form of retinal degenerative disorder. The real mechanism of interaction between rs380390 and rs2402053 should be explored further by biological experiments.

## Conclusions

While many computational methods were used for identification of epistatic interactions, most existing computational methods do not consider the complexity of genetic mechanisms causing common diseases and only focus on the selection of SNP sets, which show the best classification capacity. This will introduce many false positives inevitably. Furthermore, most existing methods cannot directly handle genome-wide scale problems. In this paper, we introduce a new and fast Markov Blanket-based method, FEPI-MB, to identify epistatic interactions. We compared FEPI-MB with three other methods, BEAM, SVM and MDR, over both simulated datasets and a real dataset. Our results show that the FEPI-MB algorithm outperforms other methods in terms of the power and sample-efficiency. Moreover, we compare FEPI-MB with one of the best Markov Blanket learning method, interIAMBnPC. The FEPI-MB is more than ten times faster than interIAMBnPC.

## Methods

### Markov blankets

Bayesian networks represent a joint probability distribution *J* over a set of random variables by a directed acyclic graph (DAG) *G* and encode the Markov condition property: each variable is conditionally independent of its nondescendants, given its parents in *G*[23]. In a Bayesian network, if the probability distribution of X conditioned on both Y and Z is equal to the probability distribution of X conditioned only on Y, i.e., *P*(*X*|*Y*, *Z*) = (*X* | *Y*), X is conditionally independent of Z given Y. This conditional independence is represented as (*X* ⊥ *Z|Y*).

**Definition 1 (Faithfulness). ***A Bayesian network N and a joint probability distribution J are faithful to each other if and only if every conditional independence entailed by the DAG of N and the Markov Condition is also present in J*[*24*]*.*

**Theorem 1**. *If a Bayesian network N is faithful to a joint probability distribution J*, *then:* (*1*) *nodes X and Y are adjacent in N if and only if X and Y are conditionally dependent given any other set of nodes.* (*2*) *for the triplet of nodes X*, *Y* , *and Z in N*, *X and Z are adjacent to Y* , *but Z is not adjacent to X*, *X → Y ← Z is a subgraph of N if and only if X and Z are dependent conditioned on every other set of nodes that contains Y .*

We can define the Markov Blanket of a variable T, MB (T), as a minimal set for which (*X* ⊥ *T*|*MB*(*T*)), for all *X* ∈ *V* – {*T*} – *MB*(*T*) where V is the variable set. The Markov Blanket of a variable T is a minimal set of variables, which can completely shield variable T from all other variables. All other variables are probabilistically independent of the variable T conditioned on the Markov Blanket of variable T.

**Theorem 2**. *If Bayesian network N is faithful to its corresponding joint probability distribution J*, *then for every variable T*, *MB*(*T*) *is unique and is the set of parents*, *children*, *and spouses of T*.

**Figure 5:**
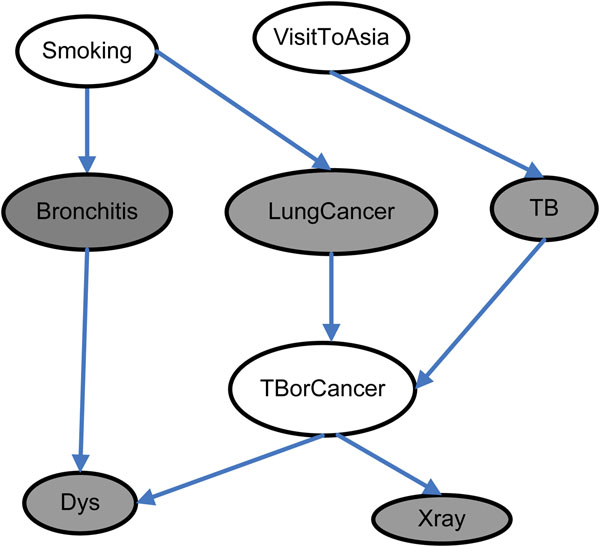
**The Aisa network.** The gray-filled nodes are the MB(T) of node ‘TBorCancer’.

**Theorem 1** and **Theorem 2** are proven in [25,26], separately. We show an example of the Markov Blanket in the well-known Asia network in Figure 5. The MB(T) of the node ‘TBorCancer’ is the set of gray-filled nodes.

Given the definition of a Markov Blanket, the probability distribution of T is completely determined by the values of variables in MB(T). Therefore, the detection of Markov Blanket can be applied for optimal variable selection and causal discovery. In this paper, we use Markov Blanket method to detect potential causal SNPs for common complex diseases.

### Markov blankets learning methods

There are several Markov Blanket learning methods such as: Koller-Sahami (KS) algorithm [27], Grow-Shrink (GS) algorithm [28], Incremental association Markov Blanket (IAMB) algorithm [15], Max-Min Markov Blanket (MMMB) algorithm [29], HITON_MB [30] and PCMB [31].

Koller-Sahami (KS) algorithm is the first algorithm to employ Markov Blanket for feature selection. However, there is no theoretical guarantee for Koller-Sahami (KS) algorithm to find optimal MB set [27]. The GS algorithm [24] and IAMB methods [15] are two similar algorithms with two search procedures, forward and backward. In the forward phase, the nodes of MB(T) are admitted into MB, while in the backward phase false positives are removed from MB. Under the assumptions of faithfulness and correct independence test, both the GS algorithm and IAMB are proved correct [15]. Comparing to GS algorithm, IAMB might achieve a better performance with fewer false positives admitted during the forward phase. A common limitation for GS algorithm and IAMB is that both methods require a very large number of samples to perform well. IAMB can be revised in two ways: (1) After each admission step in forward phase, perform a backward conditioning phase to remove false positives to keep the size of MB(T) as small as possible. (2) Substitute the backward conditioning phase with the PC algorithm instead [20]. In other words, the backward phase will perform the independence test conditioned on all subsets of the current Markov Blanket. Tsamardinos et al. proposed three IAMB variants: interIAMB, IAMBnPC and InterIAMBnPC [15]. They also proved the correctness of InterIAMBnPC. The time complexity of IAMB is O(|MB|×N) where |MB| is the size of MB and N is number of variables.

To overcome the data inefficient problem of IAMB and its variants, Max-Min Markov Blanket (MMMB) algorithm [29], HITON_MB [30] and PCMB [31] are proposed. All these three algorithms take a divide-and-conquer method that breaks down the problem of identifying Markov Blanket of variable T into two subproblems: First, identifying parents and children of T (PC(T)) and, second, identifying the spouses of T. Meanwhile, they have the same two assumptions as IAMB (i.e. faithfulness and correct independence test) and take into account the graph topology to improve data efficiency. However, results from MMPC/MB and HITON-PC/MB are not always correct since some descendants of T other than its children will enter PC(T) during the first step of identifying parents and children of T [31]. PCMB can be proved correct in [31]. In every loop, PCMB first remove unrelated variables, then PCMB use IAMBnPC method to admit one feature and remove false positives. The problem of PCMB is that the PC algorithm performs an exhaustive conditional independence test, which is very time consuming. The reason that PC algorithm was used in PCMB and interIAMBnPC is that PC algorithm is a more sample-efficient method and is sound under the assumption of faithfulness [15]. In fact if the size of Markov Blanket is large, PC algorithm still needs a lot of samples to guarantee its performance. There is no theoretical proof and guarantee that the PC algorithm admits less false positives than other methods.

### Method description: FEPI-MB

Detecting gene-gene interaction is a special application of Markov Blanket learning method because we only need to detect the parents of the target variable T and don’t need to design a complex algorithm to detect spouses of T. Here target variable T is the disease status labels and the parents of T are those disease SNPs. MB(T) only contains the parents of T.

All Markov Blanket learning methods are based on the following two Theorems.

**Theorem 3.*** If a variable belongs to MB*(*T*) *which only contains the parents of T*, *then it will be dependent on T given any subset of the variable set V-{T} .*

**Proof**: This is a direct consequence of **Theorem 1** because now MB(T) only contains the parents of T. □

**Theorem 4.*** If a variable is not a member of MB*(*T*), *then conditioned on MB*(*T*), *or any superset of MB*(*T*), *it will be independent of T.*

**Proof**: Let ***X***, ***Y***, ***Z*** and ***W*** represent four mutually disjoint variable sets. Any probability distribution *p* satisfies the weak union property: [25]. Based on the definition of Markov Blanket, we get that . Thus, by the weak union property, we have  for any subset .□

The *G*^2^ test is used to test independence and conditional independence between two variables for discrete data [13,24,32]. The null hypothesis for *G*^2^ test is that two variables are independent. As described next, the proposed FEPI-MB uses *G*^2^ to test the association and independence between SNPs and disease status.

**Figure 6:**
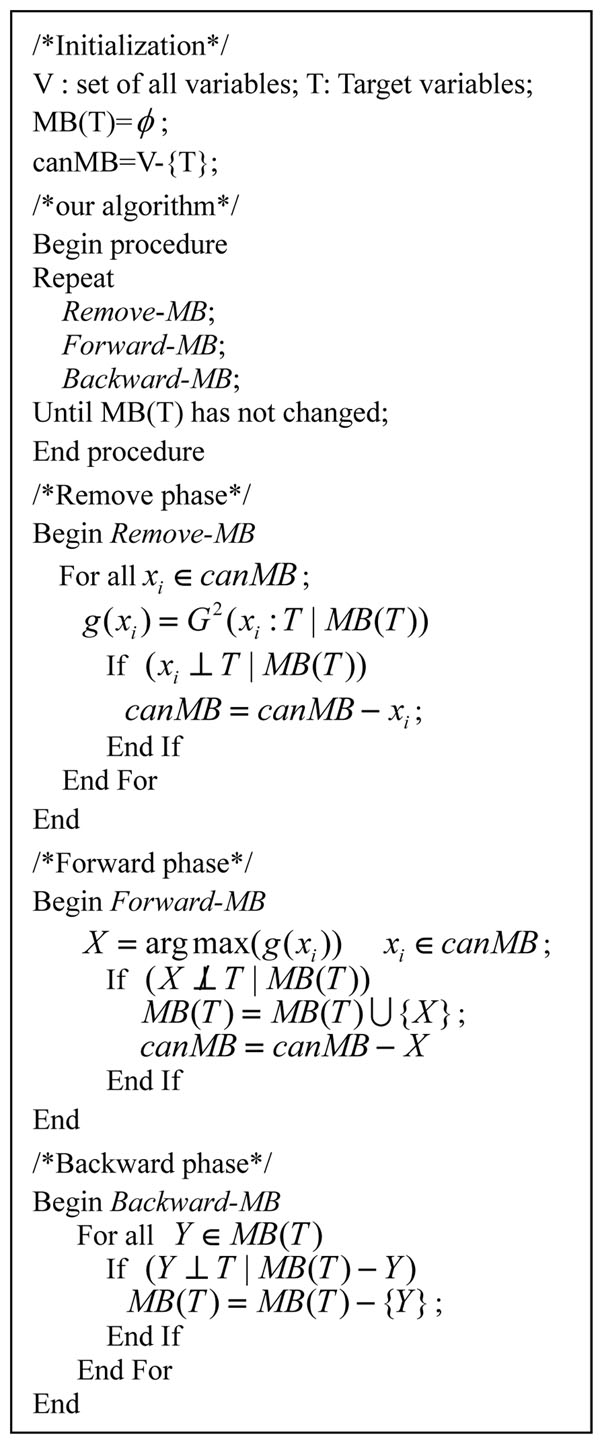
FEPI-MB algorithm.

The detail of our FEPI-MB algorithm is shown in Figure 6. It consists of three phases: *Remove*-MB, *Forward-*MB and *Backward-*MB. During the phase of *Remove*-MB, unrelated variables are removed from the candidate set for Markov Blanket (canMB) based on the conditional independence test. This will reduce the searching space after each iteration and can help to decrease the computational complexity. After the phase of *Remove*-MB, the variable which has the maximal *G*^2^ score and is associated with the target variable T in canMB enters MB(T) in the phase of *Forward-*MB, where false positives are removed during the phase of *Backward-*MB. Comparing to PCMB, we get rid of the time-consuming PC algorithm and use the maximal subset of current MB(T) to perform the conditional independence test in the phase of *Backward-*MB. The time complexity of FEPI-MB is less than the O(|MB|×N) of IAMB because in each iteration after the first iteration the number of conditional independence tests performed in the phase of *Remove*-MB is less than N. The optimal time complexity of FEPI-MB is O(N).

Like IAMB and PCMB, the soundness of FEPI-MB is based on the assumptions of DAG-faithfulness and correct independence test.

**Theorem 5**. *Under the assumptions that the independence tests are correct and that the data D are generated from a probability distribution which is faithful to a DAG G*, *FEPI-MB returns all parents of T*.

**Proof**: First, each node in MB(T) enters MB(T) in the *Forward-*MB phase and will not be removed during the *Backward-*MB phase because if , then  for any  owing to **Theorem 3**. Second, the nodes outside the MB(T) will be removed sooner or later during the *Backward-*MB phase especially after all elements in the Markov Blanket of T enter the current MB(T) because of the definition of Markov Blanket and **Theorem 4**. □

Even though FEPI-MB is a method based on the greedy algorithm, **Theorem 3** and **Theorem 4** can guarantee that FEPI-MB will not get stuck in a local optimum.

## List of abbreviations used

GWAS: genome-wide association studies; FEPI-MB: Fast EPistatic Interactions detection using Markov Blanket; SNP: single nucleotide polymorphisms; LR: logistic regression; MDR: multifactor dimensionality reduction; stepPLR: stepwise penalized logistic regression; BEAM: Bayesian epistasis association mapping; MCMC: Markov Chain Monte Carlo; SVM: Support Vector Machine; RFE: recursive feature elimination; RFA: recursive feature addition; GA: genetic algorithm; AMD: Age-related Macular Degeneration; MAF: minor allele frequencies; LD: linkage disequilibrium; HWE: Hardy-Weinberg Equilibrium; DAG: directed acyclic graph.

## Competing interests

The authors declare that they have no competing interests.

## Authors' contributions

BH designed and implemented the FEPI-MB method, tested the existing methods and analyzed experimental results. XWC conceived the study, designed the experiments, and analyzed experimental results. ZT analyzed experimental results. All authors helped in drafting the manuscript and approved the final manuscript.
